# Multi-organ involvement in preterm neonatal encephalopathy

**DOI:** 10.1016/j.earlhumdev.2025.106317

**Published:** 2025-06-09

**Authors:** Lynn Bitar, Srinivas Kota, Michelle Machie, Suleiman Mashat, Yu-Lun Liu, Lina F. Chalak

**Affiliations:** a Division of Neonatal-Perinatal Medicine, Department of Pediatrics, University of Texas Southwestern Medical Center and Children’s Medical Center, Dallas, TX, USA; b Peter O’Donnell Jr. School of Public Health, University of Texas Southwestern Medical Center, Dallas, TX 75220, USA

**Keywords:** Hypoxic-ischemic encephalopathy, Multi-organ dysfunction, Preterm, Newborn, Monitoring

## Abstract

**Background:**

Hypoxic-ischemic encephalopathy (HIE) is a complex condition resulting from oxygen deprivation at birth. As the body redirects cardiac output to protect the brain, this can lead to multiorgan dysfunction (MOD). While most previous studies traditionally focused on term neonates with HIE, our goal was to focus on preterm neonates to study the effect on organs during critical periods of brain development. We aim to assess the incidence and severity of MOD in relation to brain magnetic resonance imaging (MRI) findings and electroencephalogram (EEG) abnormalities in this vulnerable population.

**Methods:**

Retrospective cohort review of preterm neonates (<35 weeks’ gestation) with a diagnosis of neonatal encephalopathy (NE) admitted to the Neonatal Intensive Care Unit (NICU) at Parkland Hospital in Dallas between 2009 and 2023. MOD diagnosis of cardiac, renal, and liver function using clinical and laboratory markers, including echocardiography, serial troponin T, creatinine, urine output, as well as AST and ALT levels during the perinatal period.

**Results:**

During the study period, 54 preterm neonates (incidence 0.4/1000) were diagnosed with HIE. All of them had one or more organ injuries and the majority suffered from MOD: 67 % had liver injury (AST 277.0 [68.0, 686.8] IU/L), 55 % had cardiac injury (Troponin T 0.3 [0.2, 0.6] Ng/mL), and 37 % had renal injury (oliguria and creatinine 1.0 [0.8, 1.3]). Those values significantly decreased from Day 1 compared to Day 3 and 6 of life. Additionally, 35 % of newborns had electrographic seizures, 68 % had a discontinuous EEG background, and 83 % had brain MRI/MRS abnormalities supporting HIE. Death occurred in 10 (19 %) due to complications from MOD.

**Conclusions:**

By revealing the significant burden of MOD in preterm infants, this study highlights the need to refine screening and management strategies. Greater vigilance of MOD is crucial for future neuroprotective strategies in this vulnerable population.

## 1. Introduction

Perinatal hypoxic-ischemic injury is a condition in which newborns experience insufficient oxygen supply to tissues and vital organs around the time of birth [1–3]. This oxygen deprivation can significantly affect vital organs such as the brain, heart, liver, and kidneys [4–7]. While perinatal hypoxic-ischemic injury has traditionally been studied for its impact on term neonates [8], it also affects preterm neonates (born before 35 weeks of gestation) with added burden of hypoxia and immaturity.

Preterm neonates are particularly vulnerable due to a combined effect of hypoxic ischemic encephalopathy (HIE), and complications associated with prematurity, including respiratory distress syndrome (RDS), intraventricular hemorrhage (IVH), and systemic challenges such as infections and prolonged hospitalizations [9–15]. Despite these risks, defining hypoxic-ischemic injury (HI) in preterm neonates and understanding its clinical course remain complex [16–18]. While multi-organ dysfunction (MOD), involving cardiac [19–21], renal [22], and hepatic injury [23], has been extensively documented in term neonates with HIE [24], research on MOD in preterm newborns with HIE remains limited.

To address this gap, we conducted a comprehensive analysis of organ dysfunction in preterm neonates with neonatal encephalopathy attributable to HIE. Our study focused on two key objectives: (1) determine the incidence of cardiac, renal, and hepatic dysfunction, and (2) measure association between MOD, brain magnetic resonance imaging (MRI) findings, and electroencephalogram (EEG) abnormalities in this cohort.

## 2. Methods

### 2.1. Study design and population

This single-center cohort study targeted preterm neonates <36 weeks of gestation born at Parkland Hospital in Dallas, TX, between January 2009 and July 2023. Exclusion criteria were as follows: (1) neonates with significant congenital abnormalities including microcephaly and genetic conditions, and (2) those with neonatal encephalopathy attributable to other neurological conditions, such as congenital brain malformations, brain infections, intraventricular hemorrhage (IVH), or periventricular leukomalacia (PVL).

We included all preterm newborns admitted to the Neonatal Intensive Care Unit (NICU) with a diagnosis of NE. Per institutional clinical NeuroNICU protocol, the attending neonatologist documented a neurological evaluation upon admission within 6 h of birth to establish the diagnosis and severity of NE attributable to HIE [25,26]. The American College of Obstetricians and the American Academy of Pediatrics (AAP) recommends the HIE diagnosis to include: (1) the presence of a sentinel event occurring immediately before or during labor and delivery; fetal heart rate monitor patterns consistent with an acute peripartum or intrapartum event; (2) the presence of fetal acidosis and low Apgar scores, (3) evidence of multiple organ injury affecting the heart, liver, or kidneys, (4) neuroimaging with MRI consistent with acute peripartum or intrapartum event and excluding other causes [27]. Infants admitted to the NeuroNICU were identified based on institutional criteria for high-risk neonates requiring neurocritical care, independent of MRI findings alone. Importantly, the decision to admit a preterm or very preterm infant to the NeuroNICU relied on the attending physician’s clinical judgment, ensuring that neonates who would benefit most from specialized neurocritical care received timely intervention.

Neurological assessment was conducted using a modified Sarnat staging system adapted for preterm infants, acknowledging that standard scoring parameters such as level of alertness in extremely preterm neonates may be confounded by gestational immaturity. The evaluation assessed key neurological domains, including level of consciousness, spontaneous activity, posture, tone, primitive reflexes, and autonomic function, with scores ranging from 0 (normal) to 3 (severe) for each category [28]. The Total Sarnat Score (TSS) was calculated as the sum of these scores (range: 0–18), where 0 indicated normal function and 18 reflected severe encephalopathy across all six domains. For consistency, an institutional preterm-specific neurological examination form that was previously validated in preterm infants was used to ensure systematic evaluation [29]. TSS data were retrieved from electronic medical records.

EEG and brain MRI were used, when available, to help rule out alternative diagnoses and support the diagnosis of HIE. MRI abnormalities—when present—included diffuse white matter and basal ganglia involvement, as well as cortical changes indicative of ischemic injury [30]. Our standardized NeuroNICU protocol allowed testing of cardiac, renal, and hepatic injury within the first 24 h of life and serially throughout the first week.

### 2.2. Data collection

Maternal and neonatal demographics were collected through a retrospective review of medical records. Pregnancy-related characteristics included maternal age, gravidity, parity, mode of delivery, prenatal care, and the presence of clinical chorioamnionitis. Neonatal characteristics included Apgar scores at 1, 5, and 10 min, organ-specific complications, and total length of hospital stay.

MOD was assessed by evaluating three organ systems using clinical and laboratory data:

- **Cardiac function**: Assessed via echocardiography and troponin T levels. Cardiac injury was defined as troponin *T* > 0.1 ng/mL and/or echocardiographic evidence of myocardial dysfunction, wall motion abnormalities, or pulmonary hypertension [21,31].
- **Liver function**: Evaluated using aspartate aminotransferase (AST) and alanine transaminase (ALT) levels, with liver injury defined as AST or ALT >100 U/L [23,32,33].
- **Renal function**: Assessed using creatinine levels and urine output, based on the KDIGO criteria. Renal injury was defined as a rise in serial creatinine of ≥0.3 mg/dL or 50 % from the lowest previous value and/or urine output of <1 mL/kg/h averaged over 24 h on postnatal days 2–7 [6,22,34].

Biomarkers for MOD were recorded on day 1 after birth for cardiac, liver, and renal function; on day 3 for cardiac and liver function; and on day 6 for renal function.

### 2.3. Statistical analysis

To ensure an adequate sample size, and measure incidence in this inborn cohort we used a convenience sampling, including all eligible neonates documented in the registry since its implementation in 2009. To summarize maternal, neonatal, and MOD characteristics across the entire NE spectrum, we used descriptive statistics. Data were reported as mean with standard deviation, median with interquartile range (IQR), or total numbers with percentages, as appropriate. Group comparisons for continuous variables were performed using either an unpaired *t*-test (for normally distributed data) or the Wilcoxon rank-sum test (for non-normal data). Categorical variables were compared using the *χ*^2^ test or Fisher’s exact test. Associations between ALT, AST, troponin T, creatinine, and TSS were analyzed using Spearman’s rank correlation. Statistical significance was defined as a two-sided *p*-value <0.05. All analyses were conducted using R software, version 4.3.1.

### 2.4. Study approval

This study was approved by the Institutional Review Board of the University of Texas Southwestern Medical Center, Dallas, USA.

## 3. Results

### 3.1. Study population

**Fig. 1.**
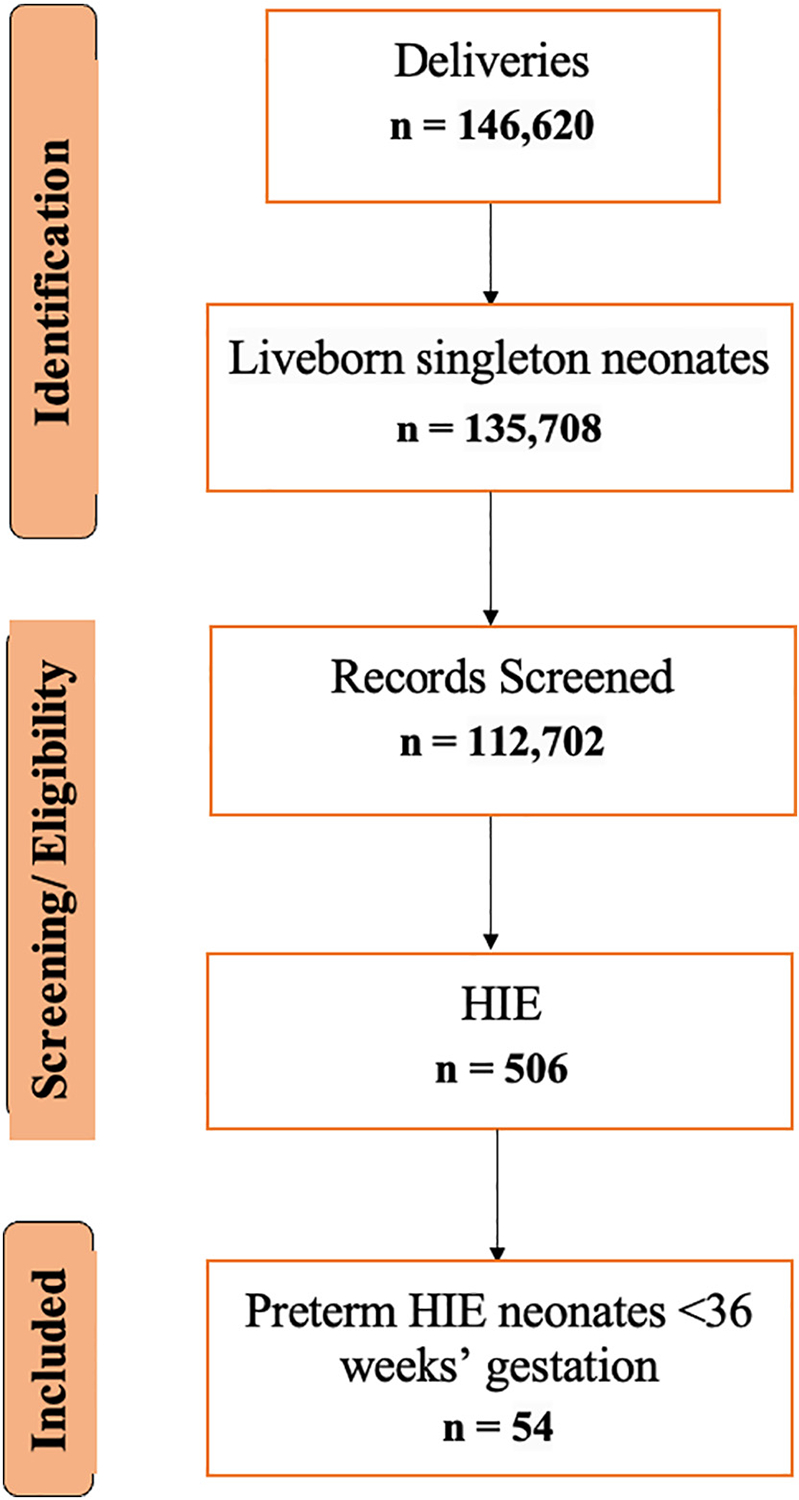
Flowchart of the study cohort selection process for preterm neonates with HIE.

Between January 2009 and July 2023, Parkland Hospital recorded 146,620 deliveries (Fig. 1). Of these, 135,708 were liveborn singleton neonates, and 112,702 inborn infants met the eligibility criteria for analysis. Among the eligible neonates, 3870 experienced metabolic acidosis or PA, and 506 were diagnosed with NE attributable to HIE at discharge. Of these, 452 were term infants (≥35 weeks’ gestation), and 54 were preterm and comprised the final study cohort.

### 3.2. Demographic, obstetric, and perinatal characteristics

Table 1 summarizes the maternal and neonatal characteristics of the cohort. The mean maternal age was 29 ± 6 years, with a median gravidity of 4 [2, 4] and parity of 2 [1, 2]. Most deliveries were cesarean sections (91 %), and 94 % of mothers received prenatal care. Only 9 % of pregnancies were complicated by chorioamnionitis. Cord arterial blood gas analysis at birth revealed a median pH of 7.15 [6.88, 7.27] and a base deficit of 13.05 [6.5, 24.78].

The median gestational age of the neonates was 32 weeks [30,33]. Among them, 69 % were White, 31 % were Black, 63 % were Hispanic, and 54 % were male. Neonates had a median Apgar score of 1 [0, 2], 4 [2, 6], and 5 [3, 7] at 1, 5, and 10 min, respectively. The median total hospital stay was 35 days [23, 65]. Neurological assessments reported a median TSS of 3 [2, 6], with blood gas analysis 1 h after resolution showing a median pH of 7.34 [7.28, 7.38] and a base deficit of 3.25 [2.0, 6.68]. Incidence of HIE was 0.4/1000 live preterm births.

### 3.3. Organ-specific biomarkers of neonates with HIE

In the cohort, liver injury was observed in 67 % (26/39) of neonates, cardiac injury in 55 % (22/40) and kidney injury in 37 % (20/54). The distribution of organ injuries varied among the neonates: 20 % experienced no organ injury, 41 % had one organ injury, 32 % had two organ injuries, and 7 % exhibited involvement of all three organs. Additionally, 61 % had either liver or kidney injury, 70 % had either liver or heart injury, and 66 % had either kidney or heart injury (Table 2).

**Fig. 2.**
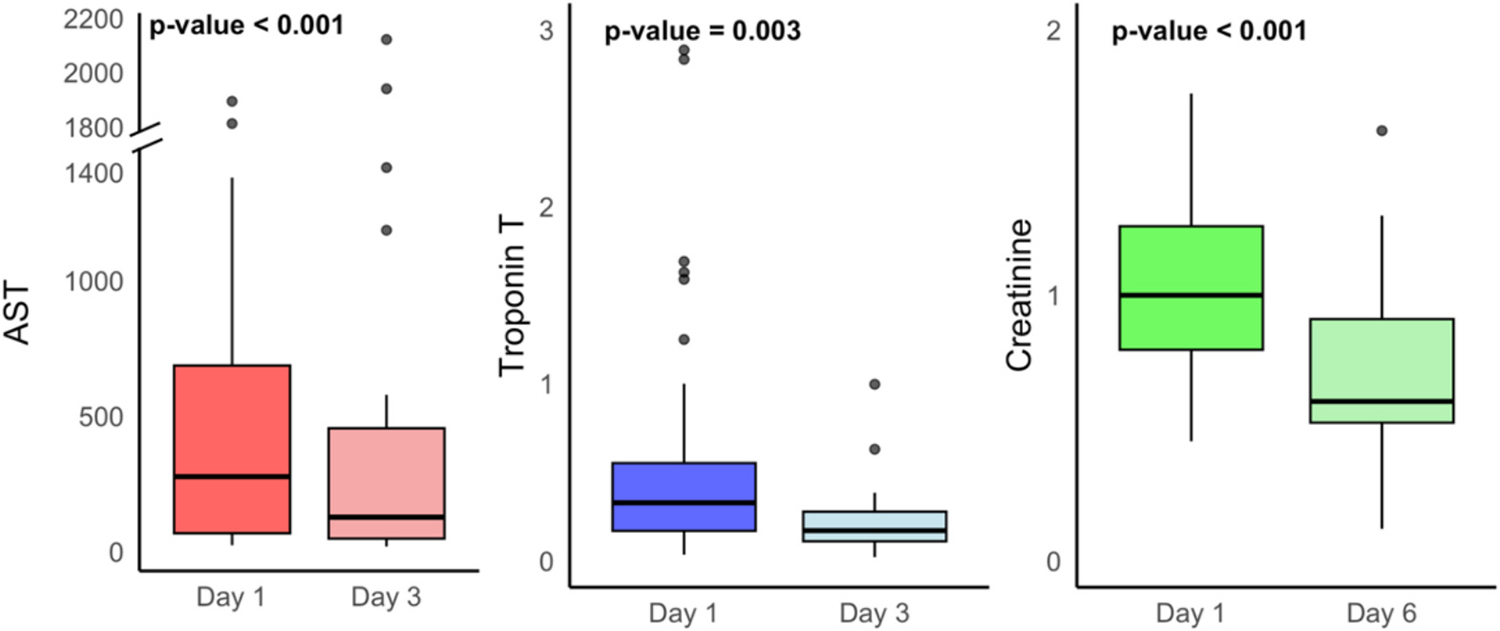
Temporal trends in biomarker levels among preterm neonates with HIE.

Biochemical markers of organ injury, assessed according to protocol, were recorded at Days 1, 3, and 6 post-birth (Fig. 2). On Day 1, the median AST level was 277.0 U/L [68.0–686.8], the median creatinine level was 1.0 mg/dL [0.8–1.3], and the median troponin T level was 0.3 ng/mL [0.2–0.6]. These levels decreased to a median AST of 127.5 U/L [48.5–455.5] by Day 3 (*p* < 0.001), median creatinine levels of 0.9 mg/dL [0.7–1.2] by Day 6 (p < 0.001), and median troponin T levels of 0.2 ng/mL [0.1–0.3] by Day 3 (*p* = 0.003).

Furthermore, 35 % (19/54) of them experienced electrographic seizures, 7 % (4/54) had clinical seizures, and 68 % (16/23) had a discontinuous EEG background. In this cohort, MRI revealed extensive abnormalities characteristic of HIE in 83 % (38/46) of cases. The MRI findings included white matter injury, basal ganglia involvement, and intraventricular hemorrhage (IVH) (Table 3). Additionally, several neonates exhibited features such as ventriculomegaly, cortical dysplasia, and global ischemic injury with MRS showing lactate peaks. Two of the four neonates with severe NE (TSS of 18) had all three organs affected. One exhibited the most significant liver injury (AST >1000), while the other had the highest troponin T (>10).

## 4. Discussion

Our study highlights the high prevalence of MOD in this vulnerable population of preterm neonates (<35 weeks of gestation). This is the first study to comprehensively explore the incidence and patterns of MOD in preterm neonates with HIE, enhancing our understanding of the relationship between organ-specific injuries and the presence of encephalopathy in this cohort.

In our cohort, nearly 80 % of preterm neonates with HIE experienced at least one organ injury, with 39 % sustaining injuries to two or more organs. While the incidence of HIE in preterm neonates (0.4 per 1000 live births) mostly involving infants born at 32 weeks of gestation was comparable to term neonates [24], the prevalence and severity of MOD in preterm neonates with HIE was strikingly high and more significant than that observed in term neonates [24,33,35]. This is likely related to immature organ systems, reduced physiological reserves, and increased vulnerability to systemic hypoxic-ischemic insults, as over one-third of brain growth occurs between 33 and 40 weeks of gestation [36]. Furthermore, organ dysfunction in preterm neonates tends to resolve more slowly, prolonging the course and impact of MOD [16].

Similar to term HIE, Liver injury was the most common, affecting 67 % of neonates, consistent with the neonatal liver’s high sensitivity to hypoxic-ischemic events due to its significant oxygen dependency [23]. Elevated AST and ALT levels are common markers of hepatic injury, reflecting the severity of hypoxic damage and often correlating with adverse outcomes [32]. Cardiac injury was observed in 55 % of the cohort, a rate comparable to that reported in term neonates with HIE but particularly concerning in preterm infants, given their increased susceptibility to myocardial dysfunction [37]. Elevated troponin levels in our cohort indicated ischemic damage to cardiac myocytes, which can lead to systemic hypotension and further exacerbate hypoxic injury to other organs, including the brain [31].

Renal injury was noted in 37 % of neonates, aligning with previous studies reporting acute kidney injury (AKI) ranging from 30 to 40 % in neonates with HIE [38]. Notably, the distribution pattern of liver, cardiac, and renal injuries in our preterm cohort parallels that observed in term neonates with HIE, suggesting a similar sequence of organ susceptibility to hypoxic-ischemic insult across gestational ages [39].

Neurological complications were significant in this cohort, with 35 % of neonates experiencing electrographic seizures. This finding aligns with prior studies on HIE, reporting electroclinical dissociation with the higher proportion in preterm neonates is likely due to their brain immaturity and vulnerability to excitotoxic injury [40]. Findings highlight the need of EEG in detecting subclinical seizure activity [41]. Furthermore, discontinuous EEG background activity, observed in 68 % of neonates, served as a marker of severe encephalopathy and poorer outcomes, consistent with prior publications [42]. Neuroimaging findings were particularly notable, with MRI/MRS abnormalities present in 83 % of neonates. These abnormalities, including elevated lactate-to-*N*-acetyl aspartate ratios, highlight the utility of advanced imaging including MRS in assessing the extent of hypoxic-ischemic brain injury and predicting outcomes [43].

The interconnected nature of organ dysfunction in HIE was evident in our cohort, with 70 % of neonates experiencing both liver and heart involvement, 67 % presenting with both kidney and heart injuries, and 7 % exhibiting injury in all three organ systems. This systemic burden underscores the need for comprehensive, multidisciplinary management strategies to mitigate complications and improve outcomes. Of note the observed mortality of 18 % in preterm was almost double that of the published contemporary mortality rate in term neonates with HIE in term neonates [44]. This likely reflects immature preterm organ development, increased susceptibility to multiorgan dysfunction, higher risk of severe brain injury, limited therapeutic options, and greater vulnerability to infection and inflammation [45].

The lack of a strong association between TSS and the extent of MOD in this cohort highlights the complex and multifactorial nature of organ injury in NE of the preterm infants. However, among the four neonates with severe NE, one infant had the highest AST and ALT levels above 1000, and death was attributable to liver failure. The remaining three neonates with severe NE exhibited varying degrees of multiorgan injury, with one affected in three organs, another in two, and the third sustained renal injury, suggesting a possible correlation between the extent of organ involvement and overall severity. Further studies are needed to explore additional contributors to MOD in this vulnerable population.

While organ injury patterns in term neonates with HIE are well-documented, data on preterm neonates remain scarce. Our findings contribute to the limited evidence available, highlighting the significant burden of MOD in preterm neonates with HIE and the critical need for early and systematic monitoring of organ function to improve survival and long-term outcomes. The use of markers such as AST, ALT, creatinine, and troponin, along with advanced neuroimaging techniques, is essential for guiding clinical interventions. Further research is needed to develop tailored interventions that address the unique vulnerabilities of this population [33].

### 4.1. Strengths and limitations

A key strength of this study is its large inborn population, with NeuroNICU databases spanning over 14 years, which facilitated identification and comprehensive analysis. The inclusion of MOD markers at multiple time points offers a unique, dynamic perspective on the progression and resolution of organ injuries in preterm neonates with HIE. Additionally, the focus on preterm neonates, a population often under-represented in HIE research, provides valuable insights into this vulnerable group.

The exclusion of neonates with congenital anomalies and myopathies, while necessary, might have led to an underestimation of the broader burden of HIE in preterm populations. Another limitation of our study is the lack of a validated neurological exam for specific to preterm infants. While Sarnat scoring has been applied in infants born at 33–35 weeks [29], its reliability and applicability in earlier preterm populations remain uncertain. This limits the interpretability of our findings in this subgroup. To address this gap, future studies should focus on developing and validating gestational age-specific neurological exam. Large NICU cohorts, such as ours, may provide a unique opportunity to systematically characterize neurological exams in preterm infants and examine their association with short- and long-term outcomes [46]. Future studies with larger and more balanced cohorts are needed to explore these differences. In addition, comprehensive evaluation of other organ systems—such as hematologic and pulmonary—may provide a more complete picture of multi-organ dysfunction in this population. Furthermore, the reliance on the selected biochemical markers, while informative, may not fully capture the complexity of organ injury or its clinical implications. While our sampling focused on the early postnatal period, we acknowledge that incorporating later time points could offer additional insights into the trajectory and resolution of multiorgan dysfunction. Most notably, measures of long-term neurodevelopmental follow-up will be critical for informing prognosis and guiding interventions in future studies.

## 5. Conclusion

Preterm neonates with HIE represent a uniquely vulnerable population with a significant risk of morbidity and mortality. This study highlights the systemic nature of HIE, revealing a high burden of multiorgan injury, with liver, cardiac, and kidney injuries being particularly prevalent. These findings highlight the urgent need for targeted management strategies tailored to the specific vulnerabilities of preterm infants. Addressing these complexities through targeted interventions and comprehensive, multidisciplinary care has the potential to significantly improve clinical outcomes and enhance the long-term quality of life for this at-risk population.

## Figures and Tables

**Table 1:** Maternal and neonatal characteristics of preterm newborns with HIE.

Maternal characteristics	All mothers (*N* = 54)
Maternal age (years)	29 ± 6
Gravidity	4 [2, 4]
Parity	2 [1, 2]
Route of delivery, N. (%)	
Vaginal	5 (9)
C-section	49 (91)
Prenatal care, N. (%)	51 (94)
Chorioamnionitis, N. (%)	5 (9)
Neonatal characteristics	All neonates with HIE (*N* = 54)
Gestational age (weeks)	32 [30, 33]
Race, N. (%)	
White	37 (69)
Black	17 (31)
Ethnicity, N. (%)	
Non-Hispanic	20 (37)
Hispanic	34 (63)
Sex, N. (%)	
Male	29 (54)
Female	25 (46)
Apgar 1 min	1 [0, 2]
Apgar 5 min	4 [2, 6]
Apgar 10 min	5 [3, 7]
Blood gas pH at birth	7.15 [6.88, 7.27]
Blood gas base deficit at birth	13.05 [6.5, 24.78]
Blood gas pH at 1 h of life	7.34 [7.28, 7.38]
Blood gas base deficit at 1 h of life	3.25 [2.0, 6.68]
Head circumference (cm)	29.75 [28.0, 31.0]
Total Sarnat score	3 [2, 6]
Birth weight (grams)	1786 [1490, 2055]
Total hospital days	35 [23, 65]
Death, N. (%)	10 (18)

For continuous variables, normality was tested using the Shapiro-Wilk test. Variables with a normal distribution are reported with the mean and standard deviation, while non-normal distributions are summarized with the median and the 25th and 75th percentiles. Categorical variables are presented with counts (N) and percentages (%).

**Table 2:** Prevalence and distribution of organ injuries among preterm neonates with a diagnosis of HIE.

Liver injury, No. (%)	26/39 (67)
Heart injury, No. (%)	22/40 (55)
Kidney injury, No. (%)	20 (37)
Total number of organs injured, No. (%)	
0	11 (20)
1	22 (41)
2	17 (32)
3	4 (7)

Cutoff values for organ injury:

Liver injury: AST or ALT > 100 U/L.

Cardiac injury: troponin T > 0.1 ng/mL and/or echocardiographic abnormalities.

Renal injury: increase in serial creatinine of ≥ 0.3 mg/dL or 50 % from the lowest previous value and/or urine output < 1 mL/kg/h averaged over 24 h on postnatal days 2–7.

**Table 3:** MRI patterns of brain injury in preterm HIE neonates.

Basal ganglia injury, No. (%)	7/46 (15)
Watershed white matter injury, No. (%)	8/46 (17)
MRS abnormal spectroscopy	4/10 (40)
Other injuries, No. (%)	
IVH	10/46 (22)
Focal bleed	6/46 (13)
Cerebellar hemorrhage	4/46 (9)
Isolated white matter injury	3/46 (7)

MRI revealed abnormalities characteristic of HIE in 83 % of cases.

## Abbreviations:

AST: Aspartate aminotransferase
ALT: Alanine aminotransferase
BUN: Blood urea nitrogen
NE: Neonatal Encephalopathy
HIE: Hypoxic-Ischemic Encephalopathy
IQR: Interquartile Range
MRI: Magnetic resonance imaging
MOD: Multi-organ dysfunction
NICU: Neonatal Intensive Care Unit
